# Almond By-Products: Valorization for Sustainability and Competitiveness of the Industry

**DOI:** 10.3390/foods10081793

**Published:** 2021-08-02

**Authors:** Marta Barral-Martinez, Maria Fraga-Corral, Pascual Garcia-Perez, Jesus Simal-Gandara, Miguel A. Prieto

**Affiliations:** 1 Nutrition and Bromatology Group, Analytical and Food Chemistry Department, Faculty of Food Science and Technology, Ourense Campus, University of Vigo, E-32004 Ourense, Spain; marta.barral@uvigo.es (M.B.-M.); mfraga@uvigo.es (M.F.-C.); pasgarcia@uvigo.es (P.G.-P.); 2 Centro de Investigação de Montanha (CIMO), Instituto Politécnico de Bragança, Campus de Santa Apolonia, 5300-253 Bragança, Portugal

**Keywords:** *Prunus dulcis*, almond skins, almond hulls, almond shells, almond blanch water, bioactive compounds, bioactivities, agri-waste management

## Abstract

The search for waste minimization and the valorization of by-products are key practices for good management and improved sustainability in the food industry. The production of almonds generates a large amount of waste, most of which is not used. Until now, almonds have been used for their high nutritional value as food, especially almond meat. The other remaining parts (skin, shell, hulls, etc.) are still little explored, even though they have been used as fuel by burning or as livestock feed. However, interest in these by-products has been increasing as they possess beneficial properties (caused mainly by polyphenols and unsaturated fatty acids) and can be used as new ingredients for the food, cosmetic, and pharmaceutical industries. Therefore, it is important to explore almond’s valorization of by-products for the development of new added-value products that would contribute to the reduction of environmental impact and an improvement in the sustainability and competitiveness of the almond industry.

## 1. Introduction

Global food supply is an ongoing concern and, above all, a major scientific challenge as population, food products, and especially waste generated continue to grow. To ensure a global food supply that complies with food safety standards and minimizes the production of residues, it is necessary to implement sustainable food systems. This paradigm leads to the search for alternative food sources without depleting the agricultural sector. One of the proposed strategies is the use of by-products derived from industrial processes with interesting applications as a source of food ingredients [1].

Nuts constitute a nutritious food with high lipid content that provide a wide range of bioactive compounds beneficial to health. They are well recognized in gastronomy for their distinguished flavor, and their consumption is recommended by nutritionists and doctors for providing antioxidants, bioactive molecules, and nutrients [2,3]. In fact, moderate doses of these foods have been shown to reduce blood levels of total cholesterol and low-density lipoprotein (LDL), both of which are linked to the development of cardiovascular diseases [4]. Several studies have demonstrated these different health properties when consumed, but there is still a need for a deeper research into the full spectra of the benefits they possess. Among these nuts, it is worth mentioning the almond [2].

**Figure 1:**
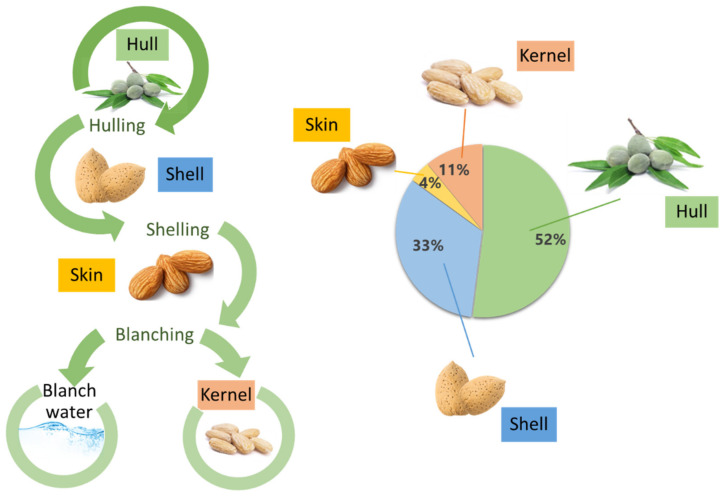
Generation of almond by-products (left) and proportion of constituents of almond fruits (right).

Almond is a stone fruit known under the scientific name *Prunus dulcis* (Mill.) D.A. Webb (herein referred to as *P. dulcis*). However, unlike other stone fruits, such as apricots or plums, where the pulp (mesocarp) is eaten and the seed (endocarp) discarded, in the case of almonds, the opposite occurs. The almond is the edible part, along the full ripening cycle of the fruit, while the mesocarp and endocarp can be eaten only at the beginning of its ripening. The main structural parts of the almond include an outer greenish cover named the hull; an intermediate shell; a brownish skin; and finally the edible seed, referred to as kernel, meat, or nut (Figure 1) [3,5]. Almond hulls have a green appearance and variable ripening cycles, which may vary due to environmental conditions such as intense heat, ultraviolet radiation, or pest infestation [3]. It is the heaviest part of the kernel, representing around 52% of the total fresh weight [6]. The common almond shell can appear in different shapes and sizes, with modifications in its appearance (wrinkles and pores mainly, among others), and its hardness is also very variable [7]. Almond skins have a brownish appearance, and it represents a light portion of the fruit with a 4% of the total weight of the almond. It is a protective layer that prevents from the oxidation and microbial contamination of the kernel [3].

As explained for other nuts, the consumption of whole almonds has been described to possess certain health benefits as it reduces postprandial glycaemia, insulinemia, and others [8]. Extracts obtained from this species have been traditionally applied for treating some diseases, neurological disorders, or respiratory and urinary tract affections. In addition, pharmacological studies indicated that *P. dulcis* has various biological activities such as prebiotic, antimicrobial, antioxidant, anti-inflammatory, anticancer, laxative, hepatoprotective, cardiometabolic protective, neotropic, anxiolytic, sedative, hypnotic, and neuroprotective effects [9].

Almond is the most produced nut worldwide in recent years. *P. dulcis* belongs to the Rosaceae family, and it is characterized for being cultivated in arid areas with dry conditions, thus motivating the establishment of sustainable irrigation practices together with good awareness to ensure high production and, at the same time, carry out an efficient development [10].

The largest almond producer is the United States, and its cultivation is generated in California in particular, with approximately 283,000 ha of cultivation [3]. According to the International Nuts and Dried Fruits Statistical Yearbook, this world production leader will have a 79% of the global almond harvest in 2020/2021, followed by Australia, in second place, which shares the 7% of the market. Spain is in third place, behind Australia and the USA, and is the country with the largest area under almond cultivation, accounting for more than 700,000 ha [11]. There is a worldwide trend towards an increase in the production of this product, which is accompanied of a parallel increase in the amount of by-products that can result in being environmentally and economically harmful if they are not properly managed [10].

The industrial process of treating almonds for consumption involves the elimination of the external parts of almond fruit in a sequential process, obtaining the almond meat or almond kernel followed by different processes: roasting, in which almond skin is detached but not eliminated, and blanching, in which the peeled almonds are generated, thus discarding the peel and generating another by-product known as blanch water [4,5]. In these processes, different forms of equipment are used to separate the meat apart from the outer part of the kernel, including shelling and peeling machines. This type of machinery is usually found in facilities close to the harvesting areas, causing less environmental impact with the transport of the product. In the final processing, almonds are packaged and stored for their distribution.

Around 10 to 25% of the total biomass that reaches the process facilities comes from the remains of the field, such as garden waste, soil, and pebbles. To these residues, one must add the by-products resulting from the dehulling process, which are separated into several piles in different areas of the facility and sorted (shell, hull, and sticks). Both hulls and sticks are predominant among all these fractions, although shells are also an important output [12]. Indeed, the processing of almonds to obtain a kilogram of nuts usually involves the production of 0.6 kg and 2.5 kg of shell and hull in terms of dry weight (dw), respectively, thus representing an annual production of shells and hulls of 0.5 and 2.2 million dry metric tons, respectively [12].

In the past, shells were used as animal feed, while the remaining almond by-products were burned for fuel production and energy use. However, almond by-products, including leaves and flowers, have been described to be of great interest as source of bioactive phytochemicals [10].

Almond hulls possess higher flavonoid content compared to other fruits. This by-product that may present variable concentration of biomolecules depending on the ripening conditions, has been demonstrated to be a rich source of triterpenoids, such as betulinic, ursolic, and oleanoic acids, as well as flavonol glycosides and phenolic acids [3]. Among its applications, almond hulls have been used as an additional source of ingredients to formulate feed diets in dairy industries, which can pay more than~$110 per ton of hulls [12].

The worldwide annual production of almond shells can account from 0.8 to 1.7 million tons. This by-product has high cellulose and lignin contents [13]. Shells can be reused in the industry itself as fuel because of its high calorific value. In detail, almond shells have been utilized during the process of scalding grains for heating water [7], and, similarly, have been exploited by other industries to generate energy as part of boiler fuel. Almond husks can also have structural functions—for instance, in the dairy industry, shells form part of the animal bedding material [12] and they can also be part of the composition of chipboard, used to polish certain metals, or used as a natural dye in wool. Nevertheless, recent reports have indicated their use as organic inclusions in ceramic bodies, as additives, or as a substrate in soilless crops [7].

Almond skins present between 70 and 100% of the total phenols present in the whole almond fruit. Many studies have demonstrated that they contain beneficial phytochemical properties, as they are a great source of phenolic compounds, such as quercetin glycosides, kaempferol, naringenin, catechin, protocatechuic acid, and vanillic acid [3,4]. In addition, polyphenols from almond skins have been shown to have synergistic effects when combined with vitamins C and E, since they protect against LDL oxidation and improve the antioxidant defense. Besides polyphenols, almond skins present different quantities of triterpenoids, especially betulinic acid, oleanoic acid, and ursolic acid, which have been described to possess anti-inflammatory, anticancer, and antiviral activity against human immunodeficiency virus (HIV).

Hence, almond by-products currently represent an issue of waste management, being potentially converted into revalorized products or ingredients in order to ensure their reduction, which would also minimize their environmental impact. In fact, as mentioned above, most of these by-products have been characterized and different bioactive properties have been identified, such as phenolic compounds that play an important role in the prevention of degenerative and cardiovascular diseases [3]. Therefore, a deeper study of the phytochemical and physicochemical composition of this fruit and by-products may allow the recovery of natural and functional ingredients with a potential application in pharmaceutical, cosmetic, or food industries for the development of innovative and sustainable added-value products.

## 2. Bioactive Composition of Almond by-Products

### 2.1. Phenolic Compounds

**Figure 2:**
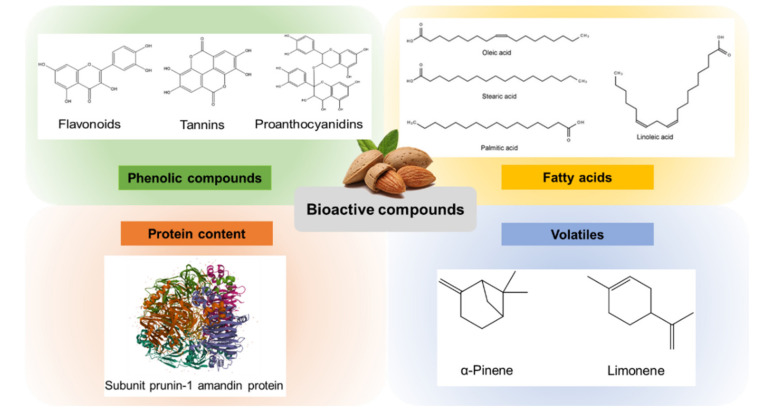
Major bioactive compounds isolated from almond and almond by-products.

Kernels of *P. dulcis* represent a rich dietary source of polyphenols that provide an important intake of condensed and hydrolysable tannins, flavonoids, and phenolic acids [14] (Figure 2). The total phenolic content of almond nuts has been found to be variable depending on the cultivar selected (Table 1) and the ripening degree, whereas the harvest year has a slight impact [15,16,17]. Several scientific works support this high content of phenolic compounds in almonds; however, the amounts are highly variable depending on the study checked. It is noticeable that the number of total phenolic compounds detected, the extraction protocol applied, the quantification units employed (determined as percentage or weight, either referred to dry or fresh biomass), the standards used for expressing final concentrations, and the detection method chosen for analyzing the phenolic content of almond kernels influence final results. For instance, in a study where 18 specific polyphenols were evaluated, these following molecules were underlined as the major ones: catechin, epicatechin, naringenin-7-*O*-glucoside, kaempferol-3-*O*-rutinoside, dihydroxykaempferol, isorhamnetin-3-*O*-rutinoside, isorhamnetin-3-*O*-glucoside, and naringenin [16]. In another assay, up to 28 polyphenols (9 phenolic acids and 19 flavonoids) were determined and their relative presence quantified. This work also points to the flavonoid catechin as a major compound (ranging from 3 to 81%, on average 46%) followed by chlorogenic acid (1–21%, 4.1% on average), naringenin (0.2–16%, 4.3% on average), rutin (0–11%, 2.1% on average), apigenin (0.1–10%, 2.9% on average), and astragalin (0–9%, 2.4% on average) [18]. While these two studies ([17,19]) employed LC–MS data for the final quantification of the targeted polyphenols analyzed, other authors [15,17] quantified the polyphenolic content using spectrophotometric techniques based on absorbance measurements, and thus they did not provide specific information on the chemical profile of the samples.

Regarding tannins, almonds have been described to contain both hydrolysable and condensed tannins. The extractable condensed tannins mostly consist of bound units of (+)-catechin and (−)-epicatechin as dimmers or trimers but also creating greater oligomers. Those can be constituted of units of (epi)afzelechin, (epi)catechin, and (epi)gallocatechin bound by A- and B-linkages that may easily reach a polymerization degree of 13, although when the polymerization is higher than six, only the B-bound is present [19]. Tannins quantified in three Californian varieties (Nonpareil, Carmel, and Butte), where the number of condensed tannins were expressed as proanthocyanidin B2 equivalents, strongly varied among varieties in a range from 322 to 1073 µg/g almond. The quantification of hydrolysable tannins provided narrower ranges for ellagic acid (487–632 µg/g) and gallic acid (141–406 µg/g) [19].

Another interesting group of polyphenolic compounds are stilbenes, although their presence in almonds is less abundant than other polyphenols. Polydatin was detected in almond kernels, almond skins, and the blanch water (0.7, 1.8, and 72 ng/g), while piceatannol and oxyresveratrol were found in almond blanch water (17 ng/g) [14].

Moreover, almond by-products such as skins have been chemically characterized and found to contain high levels of phenolic acids and flavonoids that can account for more than 30 molecular structures [14]. In fact, the variety of phenolic compounds found in almond skins included hydroxybenzoic and hydroxycinnamic acids; protocatechuic aldehydes; glycosides of flavonols and flavanones; and aglycones of flavonols, dihydroflavonols, and flavanones (Table 2). In a work based on the analysis of blanched almond skins, the authors revealed that the most abundant compounds detected belong to the flavanol class (20–38 μg of (+)-catechin per g dw of almond skin and 7–26 μg/g of (−)-epicatechin) and to the flavonol glycosides family (5–41 μg/g of kaempferol-3-*O*-rutinoside and 5–58 μg/g of isorhamnetin-3-*O*-rutinoside). These contents of phenolic compounds in blanched skins were observed to get increased after the application of dehydration treatments, such as roasting or drying. In the same work, authors also explored the presence of A- and B-type procyanidins (homopolymers of (epi)catechins), propelargonidins (heteropolymers with one unit of (epi)afzelechin, and multiple (epi)catechins) and prodelphinidin (heteropolymers with one unit of (epi)gallocatechin combined with several (epi)catechins). Procyanidins and propelargonidins were analyzed up to heptamers and A- and B-type prodelphinidins up to hexamers. Other interesting molecules found in plasma and urine as metabolites after the ingestion of almond skins were glucuronide and a few derivatives, including conjugates of naringenin and isorhamnetin, sulfate derivatives of (epi)catechin, sulfate conjugates of isorhamnetin, and conjugates of hydroxyphenylvalerolactones [4].

### 2.2. Fatty Acids

The total content of fatty acids in almonds is dependent on the cultivar, growing year, and its conditions, as it has been revealed for other biomolecules [21]. Nevertheless, different works point to similar ranges than those provided by European Food Safety Authority (EFSA), showing that the lipid content of almonds from different cultivars and locations is quite constant and in the range of 36 and 63% [19,22,23]. EFSA suggests that the fat content of almonds is around 40–50%, half of which consist of unsaturated fatty acids (UFAs), from which polyunsaturated fatty acids (PUFAs) represent 22%, mainly comprised of linoleic acid; 70% are monounsaturated fatty acids (MUFAs), with oleic acid as the major representative; and the remaining 8% is composed by saturated fatty acids (SFAs) [24]. These data are consistent with later published works that underlined that the sum of oleic (C18:1), linoleic (C18:2), palmitic (C16:0), and stearic (C18:0) acids account for over 99% of the total fatty acid content in almonds (Figure 2). The average content of UFAs, as the most abundant lipids in almonds, possess quite constant percentual values: for oleic acid (C18:1) from 58 to 77%, for linoleic acid (C18:2) 16–30%, for palmitoleic acid (C16:1) 0.2–0.6%, for vaccenic acid (C18:1) 0.7–2.2%, and for heptadecenoic acid (C17:1) 0.11%. These values demonstrate that almonds have a high amount of UFAs, whereas the presence of saturated acids is less relevant, although they also contribute to their total lipidic content. Palmitic acid (C16:0) accounts for 4.7–7.0%, stearic acid (C18:0) 1.4–2.6%, arachidic acid (C20:0) 0.06–0.12%, and myristic acid (C14:0) 0.01–0.22%. Other fatty acids present at trace levels include the SFAs pentadecanoic (C15:0), margaric (C17:0), heneicosylic (C21:0), behenic (C22:0), tricosanoic acid (C23:0), and lignoceric (C24:0) acids and the UFAs paullinic (C20:1), α-linolenic (C18:3), dihomo-γ-linolenic (C20:3), and docosadienoic (C22:2 cis 13, 16) acids [18,23].

Moreover, almonds are regarded as an important source of triacylglycerols (TAGs), since their content has been estimated to reach high percentages, about 98%. The main TAGs identified in almonds correspond to OOO (O: oleic acid) and OLO (L: linoleic acid), accounting for more than 60%. Other TAGs present in the lipidic fraction of almonds include POO (P: palmitic acid), OLL, PLO, SOO (S: stearic acid), LnOO (Ln: linolenic acid), LLL, LLP, PLP, and POP [25,26,27].

The proportion of oil in almonds is highly dependent on the ripening state of the almond kernel. The lipidic fraction becomes incremented due to a higher oil synthesis, but also because of the dehydration of the kernel, and after 14–18 weeks of maturity, almonds develop oil bodies. The maximum accumulation rate obtained for oleic acid, and thus in general for the total fatty acid content, since this is one of the major fatty acids, was between the week 14 and 17 post-anthesis (after the flower opening). Along this period of days, the maximum accumulation rate was 18 µg/day/g of fresh weight (fw), and then it decreased [26,27]. Therefore, the almond kernel development is a key phenomenon for maximizing the accumulation of lipids, both in terms of fatty acids and TAGs.

### 2.3. Volatiles

The most common volatile terpenes described in almonds are α-pinene and limonene, which have been detected in raw almonds at low levels (around 17 ng/g) [14]. Recently, different terpenes were analyzed in almond leaves, flowers, and young fruits. In leaves, the most abundant terpenes were eugenol, which reached 675 ng/g fw, and the monoterpene geraniol, for which higher recorded contents were between 31 and 54 ng/g fw. In young almond fruits, researchers quantified similar amounts for linalool (around 30 ng/g fw). The lowest amounts of terpene compounds were found in almond flowers, where other compounds like trans-linalool oxide, carvacrol, or β-cyclocitral were punctually detected but present at much lower levels (1–4 ng/g fw) [28].

Apart from terpenes, other kinds of volatiles that mainly include carbonyls, pyrazines, and alcohols had been identified in almond kernels, leaves, or flowers. Nevertheless, the most abundant amounts were described in kernels, especially after longer roasting treatments. Benzaldehyde was one of the most abundant compounds in almond leaves, where it may reach levels of 2.4 µg/g, similar to its concentration in raw almonds, around 3 µg/g. However, when raw almonds were submitted to a roasting process, benzaldehyde strongly reduced its concentration to 0.3 µg/g. Other volatiles affected by roasting were 2-methyl-1-propanol, 3-methyl butanol, 2-phenylethyl alcohol, α-pinene, and methylsulfanylmethane, whose concentration was diminished. Instead, roasting increased the amounts of other volatiles. For instance, hexanal, which was an abundant volatile in raw almonds (0.4 µg/g) and leaves (0.2 µg/g), increased its content nearly three times after the roasting of raw almonds. A similar pattern was found for 1,2-propanediol, with initial levels of 0.2 µg/g in raw almonds that got triplicated after short roasting times. The most relevant increments of volatiles after the roasting of raw almonds were found for 2- and 3-methylbutanal that increased their presence more than 100 and 400 times, respectively [28,29]. The effect of roasting on the volatile profile of almonds was also recorded on several Portuguese varieties, showing a marked increase of hexanal and benzaldehyde after roasting, whereas benzaldehyde together with 3-methyl-1-butanol were mostly reported on raw almond fruits [30].

### 2.4. Protein Content

Almonds have been described as a good source of high-quality proteins with relative amounts between 20 and 25% [24,31,32]. Regarding the protein content of almond and its by-products, the kernel, together with almond cake, possess quite abundant amounts of protein with percentages of 8.4–35% and 37%, respectively [33,34]. Other by-products have lower protein levels, such as almond skins with 10–13% [35], and almond hulls, which have the lowest values at 5.4–6.7% [36].

Almond proteins possess a chemical profile characterized to contain most of the essential amino acids, even though almonds represent a limited source of sulfur amino acids (methionine and cysteine), lysine, and threonine for children below 5 years. Instead, in adults, almonds constitute a more complete protein source since the sulfur amino acids alone are considered limiting [32].

This limiting amino acid profile is mainly provided for the major protein in almonds, amandin. Amandin is a legumin-type protein formed by two subunits, prunin-1 (Figure 2) and prunin-2, with a total molecular weight of 427 kDa, approximately. Amandin may account for nearly the 70% of total almond proteins. Regarding its amino acid profile, it is consistent with that from almonds, since its essential limiting amino acids are methionine, lysine, and threonine [31]. The majority of proteins present in almonds are hydrosoluble, and their digestibility, at least in the varieties Carmel, Mission, and Nonpareil, was shown to be higher than 82% [32]. Thus, almond and its by-products may result in an efficient source of vegetal proteins.

## 3. Biological Activities from Almond By-Products

Almond has been largely reported for its associated bioactivities, mainly focusing on the phytochemical characterization of the edible kernel. However, due to the interest regarding the valorization of almond by-products obtained along the productive workflow (mostly skin, shell, hull, and blanch water), an increasing number of studies focused on the bioactivities attributed to almond residues have appeared. Thus, the revalorization of almond by-products constitutes a promising approach during almond waste management, thus enabling the design of added-value products. Table 3 shows a general overview of the bioactivities attributed to almond and its by-products.

### 3.1. Antioxidant Activity

Thanks to their high content in polyphenols, almonds and their by-products exhibit a potent antioxidant activity, developed by different mechanisms, such as free-radical scavenging activity, antioxidant enzymes induction, modulation of genetic antioxidant response, and oxidative stress and lipid peroxidation biomarker regulation. Considering almond products, the whole seed, skins, husks, and blanch water have been mostly evaluated in terms of antioxidant activity by in vitro and biochemical assays (Table 3).

The determination of radical scavenging activity (RSA) of almond extracts have been carried out by 2,2-diphenyl 1-picrylhydrazyl (DPPH) and 2,2-azino-bis(3-ethylbenzothiazoline-6-sulfonic acid) (ABTS) free radical assays, together with the Oxygen Radical Absorbance Capacity (ORAC), and Ferric Reducing Antioxidant Power (FRAP) assays. In this sense, seven almond cultivars were subjected to the antioxidant activity determination by the DPPH and FRAP determination of flavonoid-enriched almond skin extracts [37]. Specifically, all cultivars showed high RSA rates, with values up to 90% of DPPH scavenging, whereas the Guara cultivar skins showed the highest FRAP values (556 µmol of Trolox equivalents (TE)/g almond skin). Concerning almond skin processing, drying promotes a significant increase of DPPH scavenging from 40.4 µmol TE/g of non-dried almond skins, because of the eventual Maillard reactions formed after this process [59]. In parallel, four different Italian varieties were subjected to the RSA determination of different almond by-products [38,60]. Thus, whole seeds promoted the highest rates of DPPH and ABTS scavenging activity when extracted using ethanol as solvent, whereas the rest of almond parts achieved the maximum values for hydroethanolic extracts. With respect to almond parts, the hydroethanolic skin and hull extracts of Pizzuta cultivar promoted the highest DPPH values 14.76 µg/mL [38,61], whereas the highest ABTS values were observed for hydroethanolic husk extract of Fascionello cultivar: 1.65 mM TE [38]. In the same way, the results for ORAC assay indicated that the mixture of Spanish varieties (0.5 mmol TE/g) possessed a higher activity than American varieties (0.4 mmol TE/g), being consistent with the chemical composition in terms of proanthocyanidins profile [5]. Concerning individual compounds, in terms of ABTS, DPPH, and FRAP, it was recently determined that chlorogenic acid showed the highest antioxidant activity among the polyphenols found in hydromethanolic hull extracts, whereas isorhamnetin was revealed as the most efficient antioxidant from skin extracts [62].

Besides RSA and reducing power, almond extracts have been assessed in terms of antioxidant enzyme induction, especially aqueous skin extracts, thanks to their high proanthocyanidin content, as demonstrated by the promotion of glutathione peroxidase (GPx), catalase (CAT), and superoxide dismutase (SOD) activities, attributed to proanthocyanidins concentrations of 25–50 µg/mL [40]; additionally, the hydromethanolic leaf extracts of the Mazzetto cultivar were shown to induce ascorbate peroxidase (APX) activity [41]. Furthermore, the same extracts were shown to modulate the in vitro expression of signaling pathways, by boosting the activation of nuclear factor-E2-related factor 2 (Nrf2) and promoting the expression of antioxidant response element (ARE), both involved in the cellular antioxidant system [40].

In addition, blanched almond skins have been reported to modulate plasma biomarkers of oxidative stress, such as GPx activity, glutathione concentration and the ratio reduced glutathione/oxidized glutathione [42]. According to a recent study, the antioxidant properties of polyphenol-enriched hydroethanolic almond hull extracts as inhibitors of the toxicity caused by the induced oxidative stress in Caco-2 cancer cell line was reported as a result of reactive oxygen species (ROS) scavenging, and the regulation of cell redox status [43]. Indeed, these oxidative stress-alleviating properties of almonds have been proved by in vivo human trials, indicating that almond consumption prevents the oxidative DNA damage and lipid peroxidation in male smokers [63]. Besides DNA oxidation prevention, ethanolic hull extracts prevented protein oxidation [42]. Moreover, the inhibition of lipid peroxidation of almond peels has been also assessed in vitro [42,64], together with the methanolic almond fruit extracts of different Portuguese varieties [17], by means of the inhibition of peroxidation damage in biomembranes, through the thiobarbituric acid reactive substances (TBARS) formation, and the inhibition of the induced oxidative hemolysis in erythrocytes, mostly developed by Duro Italiano cultivar. Overall, the positive health effects, in terms of antioxidant activity, associated with the consumption of almonds could be partly due to the almond skin phenolic composition, predominantly containing proanthocyanidins and flavonols, that could be used as an added-value by-product to be exploited as dietary antioxidant ingredients [3].

### 3.2. Anticancer Activity

As observed for antioxidant activity, the anticancer properties associated with almond and its by-products has been assessed by both in vitro and in vivo studies (Table 3), as a consequence of the plethora of bioactive compounds found on these matrices, especially polyphenols; acid-soluble polysaccharides; triterpenoid acids (such as ursolic, oleanolic, and betulinic acids); and UFAs proceeding from different almond seed-associated products, including kernel, skins, hulls, and oil [9]. Nevertheless, the anticancer effects attributed to almond are closely related to those of antioxidant activity, since oxidative stress is considered one of the major process involved in the early stages of carcinogenesis [49].

Taking this into account, the acetonic almond seed extracts, with high concentrations of phenolic compounds, essentially containing phenolic acids and flavonoids, showed high antiproliferative effects on two different human breast cancer cell lines, MCF-7 and MDA-MB-468, exhibiting effective cytocidal concentrations at 10 µg/mL and >20 µg/mL, respectively [44]. In the same way, the aqueous bitter almond kernel extracts showed an impressive cytotoxicity in a dose-dependent manner against MCF-7 and human colon carcinoma cancer (HCT-116) cell lines, whereas the methanolic extracts of the same matrix promoted the highest cytotoxicity against human hepatocellular cancer cell line (HepG2), showing growth inhibitory concentrations (GI_50_) of 29.5 µg/mL, 24.5 µg/mL, and 10.1 µg/mL, respectively [45]. As a matter of fact, the cytotoxicity attributed to polyphenols from methanolic almond kernel extracts against MFC-7 and HepG2 cancer cell lines was proved to be due to the induction of cell cycle arrest at the G2/M phase, associated with preG1 apoptosis induction, via the coordinated upregulation of cyclin-dependent kinase inhibitor 2A (CDKN2A) and the inhibition of cyclin-dependent kinase 4 (CDK4) genes [50]. Besides the kernel, the almond hull has also been reported as a polyphenol-enriched by-product, whose the hydroacetonic extracts showed a potent cytotoxicity against human osteosarcoma cell line (Saos-2), exhibiting GI_50_ values = 123.7 µg/mL, mostly affecting cancer cell cycle progression, which was arrested at G2/M phase, impairing the mitochondrial function, inducing caspase apoptotic activity, and inhibiting tumoral cell migration [46].

In addition to almond-derived polyphenols, UFAs detected in the almond seed oil, mostly oleic and linoleic acids, also promoted an intense antiproliferative effect on two colon carcinoma cell lines, either primary (Colo-320) or metastatic (Colo-741), in a dose- and time-dependent manner [47]. Moreover, in this particular study, almond seed oil was reported to inhibit cancer cell invasiveness, by the downregulation of bone morphogenetic protein 2 (BMP-2) and β-catenin pathways, and growth, by the inhibition of Ki-67 (a marker of cell proliferation) expression. In the same way, different polysaccharidic fractions from ethanolic almond skin extracts, mainly composed of arabinose, galactose, and mannose, promoted a cytotoxic effect on both human colon carcinoma Caco-2 and murine melanoma B-16 cell lines, with proliferation inhibition ratios of 88.74% and 90.96%, respectively [48]. Finally, terpenoids from ethylacetate Sicilian almond hull extracts were assessed in terms of their antiproliferative properties, specifically betulinic acid, which exhibited an excellent cytotoxic activity toward MCF-7 (GI_50_ = 0.27 µM), even higher than that of the anticancer drug 5-fluorouracil [65,66]. On these bases, both almond and its by-products can be considered as a new source of pharmaceuticals in the management of cancerous tumors of different origins.

### 3.3. Anti-Inflammatory Activity

Due to the close relationship of inflammation on oxidative stress and carcinogenesis, the determination of anti-inflammatory effects on almond and by-products is essential to provide an added value to these food products. Consequently, the high concentrations of bioactive compounds found on these matrices, mostly polyphenols, UFAs, and protein hydrolysates, are considered the major responsible for this bioactivity. Thus, there is a large amount of scientific evidence about the multifaceted anti-inflammatory effects of almond and by-products, as reported by in vitro, in vivo, and interventional studies in humans, acting as inhibitors of inflammatory enzymes, pro-inflammatory cytokine (CK) production, oxidative stress, and inflammatory marker-lowering agents (Table 3).

In this sense, the in vitro anti-inflammatory properties of almonds have been mostly recorded in lipolysaccharide (LPS)-induced RAW264.7 macrophages cell line. The oleic acid-enriched oily almond extracts promoted the inhibition of the inducible nitric oxide synthase (iNOS), and cyclooxygenase-2 (COX-2), by reducing the levels of the inflammatory mediator tumor necrosis factor alpha (TNF-α) [52]. Furthermore, the same extracts promoted the reduction of pro-inflammatory CKs levels, mostly intereleukin-1β (IL-1β) and IL-6, as well as the mediator nitric oxide (NO) levels. Such effects were in accordance with those observed for pepsin hydrolysates obtained from almond flour [67] and those provided by acetonic dried almond skin extracts from the Corrente/Tuono mixture variety on LPS-induced intestinal IEC-6 cell line, together with the inhibition of oxidative stress, via ROS levels reduction [68].

In parallel, the in vivo almond-associated anti-inflammatory effects have been assessed using different rodent models. In particular, the polyphenol-enriched Marcona almond blanch water was applied to in vivo rat colitis models, causing a reduction in inflammatory cell infiltrate, together with the inhibition of myeloperoxidase (MPO) activity and other related enzymes, such as iNOS [51]. In addition, the same study promoted an inhibition of oxidative stress, together with a reduction in pro-inflammatory CKs, including IL-1β, cytokine-induced neutrophil chemoattractant 1 (CINC-1), and monocyte chemoattractant protein-1 (MCP-1). Accordingly, another colitis-induced mice model fed natural almond skin powder exhibited similar results on the prevention of intestinal inflammation, modulating the associated nuclear factor κB (NFκB) and c-Jun N-terminal kinase (JNK) signaling pathways, inhibiting enzymes such as iNOS and poly(ADP-ribose) polymerase (PARP), and decreasing the levels of leukocyte-activating markers intercellular adhesion molecule (ICAM-1) and P-selectin [55]. Besides such colitis in vivo models, the ethanolic almond seed extracts were applied to renal cell carcinoma-induced rat models, demonstrating an inhibition of the cancer-related inflammatory process by decreasing pro-inflammatory CKs (IL-1β, IL-6, and TNF-α) levels, together with those of several inflammatory mediators (prostaglandin E2, PGE2, and NFκB) in a dose-dependent manner [54].

Finally, the effectiveness of almond and its by-product consumption as anti-inflammatory agents has been reported by different interventional human trials, as indicated by the reduction of circulating levels of C-reactive protein (CRP) and E-selectin inflammatory mediators, mostly guided by the high concentration of UFAs as part of almond-containing diets [69]. Consequently, these results facilitate the characterization of almond as a natural source of anti-inflammatory compounds, although further studies regarding the characterization of the mechanism of action of such compounds are required.

### 3.4. Antimicrobial Activity

Recent studies have pointed at the polyphenols from almond and its by-products as the major antimicrobial agents found on these matrices, which has been reported for different bacterial, fungal, and viral species (Table 3) [9]. In fact, the antimicrobial activity of almond has been assessed, indicating its high diversification, especially attributed to skins, but also hulls and blanch water [39].

The antibacterial effects of almond skin extracts have been largely assessed for a wide range of both Gram-positive and Gram-negative bacteria. Thus, polyphenol-enriched aqueous methanolic extracts from almond skins and hulls were subjected to antibacterial assays, revealing that skin extracts exhibited significantly higher antibacterial activity rates, even greater than those of antibiotics such as gentamicin, against *Pseudomonas aeruginosa*, *Staphylococcus aureus*, *Enterococcus faecalis*, and *Listeria monocytogenes* [10]. The same authors suggest the high concentrations of phenolic compounds as the cause of this antimicrobial activity, mostly due to naringenin, (-)-epicatechin, protocatechuic acid, catechin, and isorhamnetin-3-*O*-glucoside. Equally, the methanolic skin extracts of Pizzuta variety showed that blanching promotes a significant loss of the antimicrobial activity, which partially remains in the blanch water [64]. On these bases, these results were in line with other works, in which the bacteriostatic effects of methanolic peel extracts were reported for *Salmonella enterica* var. Typhimurium, *L. monocytogenes*, and *Staphylococcus aureus*, due to the high concentrations of epicatechin, protocatechuic acid, and naringenin [35]. Moreover, the same extracts have been seen to exert a potent inhibition in the proliferation of *Helicobacter pylori* from different clinical isolates, referring to protocatechuic acid as the major responsible for this effect [53]. Due to the wide evidence regarding the antibacterial properties of almond by-products, recent reports have proposed the application of almond gum for the design of antimicrobial zinc oxide nanoparticles with a potent antimicrobial activity against *S. aureus*, *Escherichia coli*, and *Salmonella paratyphi*, as well as antifungal activity against *Candida albicans* [70].

Besides polyphenols, polysaccharides from almond tree gum, mostly containing galactose, arabinose, xylose, mannose, rhamnose, and glucuronic acid as monosaccharide residuals, were explored in the basis of antimicrobial activity, being effective bacteriostatic compounds toward *S. aureus*, *P. aeruginosa*, *S.* Typhimurium, *E. faecalis*, and *E. coli* [71]. Moreover, hemicelluloses from the same almond gum have been seen to promote a higher antibacterial activity than polysaccharides in the case of both Gram-positive species, such as *Bacillus subtilis*, *Actinomyces* sp., and *S. aureus*, and Gram-negative species, such as *Klebsiella pneumoniae* and *Salmonella typhi* [72]. Such results provide insight about the consideration of almond by-products for their revalorization, not only associated with almond fruits, but also the products obtained from almond trees.

In addition to the antibacterial and antifungal properties of almond by-products, their polyphenol-enriched derived extracts have been investigated to determine their antiviral activity. For this purpose, methanolic peel extracts were applied to herpes simplex virus-1 (HSV-1)-infected Vero cell lines, promoting an antiadhesive activity of HSV-1, thus preventing its introduction into the cells, suppressing the synthesis of early viral proteins and the accumulation of viral DNA [39], and increasing the virus exposition to the action of polyphenols, mostly due to the high concentration of flavonones [73]. In parallel, the aqueous peel extracts developed a potent antiviral activity against HSV-2 at extract concentrations of 60 µg/mL, mainly guided by kaempferol glycosides, which were responsible for boosting the immune response in peripheral blood mononuclear cells by modulating the synthesis of different CKs, such as interferon gamma (IFN-γ), IL-4, IL-10, and TNF-α [74]. Due to the multifaceted antimicrobial properties of almond skins, further studies should be aimed at characterizing the mechanisms of action of these natural antimicrobials, as well as the potential synergism between molecules on the treatment of pathogenic bacterial, fungal, and viral infections.

### 3.5. Prebiotic Activity

Almond and its by-products have been valorized as prebiotic products, as well, especially almond skins, promoting the enhancement of intestinal microbiota diversity and improving the overall gastrointestinal function. In this sense, almond consumption including skins has been reported to increase β-galactosidase activity, considered as a marker of beneficial colonic bacteria, such as bifidobacterial and lactobacilli, thus improving carbohydrate metabolism of chronic ailments, such as Crohn’s disease and ulcerative colitis (Table 3) [9].

The prebiotic activity of almond peels was reported by an in vitro digestion model, showing that the dietary fiber derived from this by-product enhanced the bifidobacterial population, including *Clostridium coccoides* and *Eubacterium rectale* after a 24-h incubation period [75]. In addition, the same group demonstrated that both bacterial groups were not negatively affected by the presence of polyphenols in this matrix, and that butyrate production played a critical role in the enhancement of their growth [75], as a consequence of the metabolization by the gut microbiota of UFAs present in almond and its by-products [56]. Such butyrate-mediated bifidobacterial promotion was assessed by Rocchetti et al. (2019), suggesting that polyphenols from almond seeds are catabolized by colonic bacteria during fecal fermentation [57]. Thus, skins constitute a prominent by-product causing the prebiotic effects of almonds, thanks to their high content of dietary fiber, representing the 45% of their weight [75], and a source of bioactive prebiotic molecules, as it is the case of xylooligosaccharides (XOS), polysaccharides, and hemicelluloses [76,77].

Besides the in vitro assessment of prebiotic activity, different clinical interventions in humans have shed light about the effect of almond skin consumption on the gut function, revealing that both the *Bifidobacterium* spp. and *Lactobacillus* spp. increased their populations in fecal samples, whereas it suppressed the multiplication of the pathogenic *Clostridium perfringens* [58]. Moreover, the almond roasting process slightly decreased the prebiotic effects of almonds in comparison with the natural ones, although it improved the metabolic effects at the intestinal tract [78]. In another randomized controlled trial, a positive correlation between almond consumption and gut microbiota promotion was reported, indicating that chopped almonds intensified *Lachnospira*, *Roseburia*, and *Oscillospira* growth, whereas whole almonds increased the *Dialister* populations [56]. As a result, the prebiotic effects of almond skins have generated an increasing interest in the inclusion of this by-product in functional foods, as it is the case of functional biscuits, whose nutritional properties were improved in terms of fiber and phenolic compound content [79], revealing a promising applicability of almond by-products in the inclusion of functional ingredients for the food industry.

### 3.6. Other Activities

Besides the above-mentioned bioactivities attributed to almond by-products, which have been largely assessed, there are additional functionalities reported on these matrices that open new perspectives for their valorization as source of functional ingredients. In this regard, different authors have highlighted the effectiveness of almond by-products on a range of chronic diseases, including dyslipidemia, diabetes, and cardiovascular diseases (CVD), as well as their role as neuroprotective and hepatoprotective agents (Table 4).

In the case of dyslipidemia, a great variety of interventional studies have pointed at a beneficial impact of the consumption of almond and by-products on lipid metabolism. Indeed, the daily consumption of almonds has been seen to promote a reduction of total cholesterol (TC), low-density-lipoprotein cholesterol (LDL-C), and apolipoprotein B, without interfering with high-density-lipoprotein cholesterol (HDL-C) [9]. This improvement of lipoprotein profile is accompanied by a reduction in TAGs levels, mostly motivated by the presence of UFAs, minerals, vitamins and phytosterols in almond and by-products [80]. In addition, polyphenol-enriched skin extracts, used as fortifier ingredients of milk, were reported to delay LDL-C oxidation in healthy adults, by promoting the increase of plasma catechin and naringenin levels [42]. Consequently, these effects promote a reduction in the central body adiposity, together with body weight and body mass index reduction, which have been linked to the amelioration of metabolic syndrome, obesity [77,83], and CVD by the reduction of atherogenic lipids levels and blood pressure [80,81].

Concerning the antidiabetic effects of almond, a number of interventional studies have assessed its positive effects, being considered a food with low glycemic index, although the identification of the constituents involved in such effects still remains a challenge [77]. In this sense, several authors have suggested that UFAs and fiber may be the major responsible molecules, developing a glycemic control by reducing blood glucose level through the stimulation of glycoprotein 1 (GLP-1) production [9,82] and carbohydrate absorption, as well as improving insulin sensitivity in type-2 diabetic patients [83,84].

Besides the cardioprotective effects, almond by-products have been also characterized by their effectiveness as protectors of hepatic and neurological functions. The hepatoprotective effects of almond skins have been demonstrated in hepatotoxicity-induced in vivo models, showing that procyanidins-enriched extracts decreased the serum levels of both hepatic alanine aminotransferase (ALT) and aspartate aminotransferase (AST), as well as improving the levels of antioxidant enzymes in the liver, including GPx, SOD, and CAT [40]. Such effects found in vivo were recently assessed in a randomized controlled clinical trial, showing that almond consumption provoked a significant reduction in serum ALT, AST, and gamma-glutamyl transferase (GGT) after 12 weeks, thus promoting an effective liver protection in patients with coronary artery disease [85].

## 4. Current Trends and Future Perspectives

Currently, consumers′ habits are more selective, which has switched the tendency of productive systems into the implementation of more efficient and environmentally friendly production practices. As mentioned above, almond is one of the most important nuts both in terms of surface cultivation area and production worldwide. The agri-food by-products can be used as an alternative source of natural ingredients that may be applied for the development of high added-value products for various industries, facing the production of food, new materials, and energy. As a result, valorization is key factor to provide a proper waste management, leading to their minimization and the consequent establishment of a more integrated and sustainable industrial system, based on the implementation of the circular economy model, including both food-related and non-related sectors. This approach has been regarded as a high-throughput productive system for being able of transforming wastes into profitable products, hence providing both environmental and economic benefits.

### 4.1. Almond and Its By-Products in the Food Industry

Due to its health benefits, in the last recent decades, almond consumption has increased, as it is a food rich in nutrients associated with health benefits. Therefore, the increment of the almond market size has been accompanied of a larger generation of related by-products [87]. Among the multiple potential applications of almond by-products, the most common ones developed have been mostly focused on biomass generation for their further use in the food and feed industries.

As a consequence of the heterogeneous products derived from almond production systems, almond market is segmented according to different factors: (i) by type—the almond market is divided into “Shelled Type”, in which shells are removed from almond fruits, and “In-shell Type” [88], and (ii) by application—the almond market is divided into direct edible use, food processing, and kitchen ingredients [89]. Besides the already mentioned health-promoting of almond kernels, several derived products are gaining a significant relevance in the food industry, especially almond milk and almond oil. Almond milk constitutes an excellent alternative to cow’s milk, mostly motivated by the increasing emergence of milk allergy and lactose intolerance among the general population. In this sense, this almond-based product experienced a fast-growing presence in both North American and European markets due to its high content in MUFAs, which assist in body weight management and lowering LDL-C levels [90]. In the same way, almond oil, characterized by their high MUFA content, has been also reported because of its beneficial impact on cardiovascular disease prevention, acting as an enhancer of blood lipoprotein profiles [91]. Moreover, the use of partially delipidified almond flour (PDAF) obtained from the extraction of almond oil is an example of the use of this by-product as an ingredient for the manufacture of biscuits under the name of “almendrados” [1].

As a result, almond by-products represent valuable residues, as they represent a promising source of sustainable and natural ingredients that may have further applications in the food industry.

### 4.2. Other Uses for Almond By-Products

Besides the importance of almonds and derived products for food applications, these resources have been explored for novel approaches, in order to implement a solid circular economy system around this productive agri-food sector. Among them, almond shells play a central role in this purpose due to their lignocellulosic nature, which provides a wide range of applications associated with the exploitation of this material.

One of the alternative applications that are still in progress is the use of almond shells as heavy metal adsorbents in the wastewater, mainly from the textile industry. The presence of heavy metals in wastewaters is a great environmental problem because of their pollution effects to either superficial or ground waters [3]. Nowadays, the search of different adsorbents to these pollutants is focused on the use of various natural and low-cost materials recovered from by-products. Scientific literature provides a vast variety of cost-effective materials that has been used as adsorbents, such as tannin-rich materials and lignin obtained from pine bark, dead biomass, or sawdust; peat moss; chitin and chitosan from fly ash; modified wool and cotton; rice husks; or animal bones [3]. Almond shells and other similar agricultural by-products, such as sugar cane bagasse or walnut shells, have been pointed out as an economic and rich source of biomass for the preparation of activated carbon. Most of the materials used for activated carbon preparations are not self-renewing. Therefore, the use of almond by-products shows a double environmental benefit. The high added-value ingredient recovered as activated carbon is aimed for adsorption, separation, and purification processes to perform in aqueous or gaseous solution systems. Activated carbon can also be used in catalytic processes, acting itself as a catalyst and thus playing an important role in different chemical, pharmaceutical, and food industries [92,93]. Few studies have analyzed the adsorption capacity of almond shells as an environmentally friendly and low-cost adsorbent on several textile dyes. Different approaches that considered the type of shell, pH, or various activated carbon formulae have been developed for removing various textile dyes (Direct Red 80, methylene blue or crystal violet) from wastewaters. In general terms, the application of almond shells results in a very efficient dye removal that may reach values of 97% that imply dye retentions of nearly 50 mg/L for Direct red 8, 148–833 mg/g for methylene blue, or 625 mg/g for crystal violet [92,93,94]. Therefore, although the generation of industrial effluents containing textile dyes is almost inevitable, both previous and future experimental studies may provide suitable remediation plans for the minimization of their unfavorable impacts by the elimination of secondary materials from these wastewaters [92].

In addition, the lignocellulosic composition of almond shells has motivated their application for the production of wood-based composites, but also prompted the fortification of plastic materials [95,96]. Concerning other industrial uses with promising results, the high lignin content of shells was correlated with a high heating value, indicating the possible use of this almond by-product as fuel [97]. Moreover, with respect to the agricultural sector, almond shells have been identified by as natural growing media for soilless crop culture, thus being considered as a potent solution to develop their use as an ecological and environmentally friendly substrate, as already demonstrated for tomato culture [98].

On the other hand, almond milk and oil have been reported for their potential use on cosmetics, being valuable skin and hair-care products administered topically with the aim of promoting healthy effects, such as soothing; pain-relief; circulation-enhancement; moisturizing; and in the case of oil, sunlight protection [99]. Consequently, the multifaceted nature of almond and derived products facilitate their exploitation on different economical and industrial sectors, simplifying the establishment of a circular economy system, involving not only the food industry, but also animal feed, cosmetics, pharmaceutical, and material industries.

## 5. Conclusions

The current increasing trend of almond nut production has been accompanied with an increment in the waste generation, which reveals the importance of seeking solutions to minimize and reutilize them. Traditionally, studies have mainly focused on the phytochemical characterization of almond nuts. However, currently, numerous studies analyze the bioactivities associated with different biomolecules recovered from the almond by-products: shells, skins, hulls, and blanch water. They are considered to have a high content of phenolic compounds, but in very variable quantities depending on various factors, such as the crop selected, the ripening time, the extraction protocol, and the quantification method used. In almond skins, high levels of phenolic acids, including both hydroxybenzoic and hydroxycinnamic acids and flavonoids, are mostly represented by naringenin and catechins. Other compounds described as characteristics of almond by-products are tannins, which can be found both as hydrolysable and condensed forms. As for the total fatty acid content, previous studies have shown that 99% of the fatty acids in almonds are composed of oleic, linoleic, palmitic, and stearic acids, revealing that MUFAs are the most prevalent compounds. Additionally, almonds and their by-products can be an effective source of vegetable proteins, as they have a high percentage (20–25%) in both the kernel and the almond cake. However, almond shells and skins have lower protein levels with 10–13% and 5–7% values, respectively.

The high polyphenol content, both in almonds and their by-products, confer to these natural matrices a potent antioxidant activity related with positive health effects. Regarding by-products, almond shells contain high levels of proanthocyanidins and flavonols with strong antioxidant capacity associated. Besides polyphenols, the presence of other bioactive compounds such as UFAs and high-quality proteins reinforces the antioxidant capacity of almond by-products and prompts their ability as anti-inflammatory and even anticarcinogenic agents. In parallel, the presence of polyphenols in by-products has been described to be capable of acting as antimicrobial agents, being effective against both Gram-positive and Gram-negative bacteria, fungi, and viruses. On the other hand, almond shells can provide prebiotic activity as they may promote the diversification of gut microbiota and improve the gastrointestinal function. In addition to those mentioned above, there are additional functionalities of these matrices that serve as prospects for their valorization as a source of functional ingredients. Indeed, the efficacy of some almond by-products has been proved in several interventional studies to be effective on the prevention of chronic diseases, such as dyslipidemia, diabetes, and cardiovascular diseases, as well as their role as neuroprotective and hepatoprotective agents. Moreover, almond by-products have been described to be applicable to industrial fields such as wastewater treatments, and the production of active carbon, fuel, and heating energy.

In summary, the application of the circular economy model to the exploitation of almond by-products may allow the recovery of natural ingredients to prompt the formulation of new nutritional, cosmetic, and pharmaceutical products, a new productive strategy that would meet future consumer expectations on environmental impact and human health.

## Figures and Tables

**Table 1:** Total content of polyphenols of different almond varieties from diverse geographical origins (expressed as mg of gallic acid/g dw).

Geographical Origin	Almond Variety	Phenolic Content (mg/g)	References
Australian	Johnston Prolific	0.8–0.9	[15]
Californian	Butte	6.1	[16]
	Carmel	6.1	
	Fritz	3.6	
	Mission	6.1	
	Monterrey	4.3	
	Nonpareil	5.3	
	Texas	1.5	[15]
	Thompson	0.8–1.5	
Italian	Duro	164	[17]
	Filippo Ceo	1.3–2.7	[15]
	Genco	2.2–11.0	
	Tuono	1.3–4.9	
Spanish	Desmajo Largueta	1.7–1.8	[15]
	Marcona	0.3–1.0	
Italian and Spanish	Ferragnès	2.5–4.2	[15]
	Francolì	2.7–7.3	
Portuguese	Pegarinhos	34.2	[17]

**Table 2:** Phenolic content in almond products and by-products. Quantitative data extracted from [4,14,20] ^1^.

Compounds	Blanched Skin (μg/g)	Whole Almonds (μg/g)	Blanch Water (μg/g)
**Hydroxybenzoic acids and aldehydes**	**26.3–57.3**	**2.4–7.0**	
p-Hydroxybenzoic acid	3.1–7.2	0.03–0.06	
Vanillic acid	11.1–19.2	1.1–2.5	
Protocatechuic acid	6.7–17.2	1.3–4.4	
Protocatechuic aldehyde	5.4–13.7	-	
**Hydroxycinnamic acids**	**1.8–4.9**	**-**	
*trans* p-Coumaric acid	0–1.1	-	
3-O-Caffeoylquinic acid	1.8–3.8	-	
**Flavan-3-ols**	**61.5–134**	**12.7–51.3**	
(+)-Catechin	20.1–38.3	9.5–38.6	
(−)-Epicatechin	7.2–26.5	3.2–12.7	
Procyanidin B1-3, B5, B7, C1	25.0–46.1	-	
Unknown dimers/trimers A [(epi)catechin → A → (epi)catechin]	9.2–23.0	-	
**Flavonol glycosides**	**15.6–130**	**113–231**	
Kaempferol-3-O-rutinoside	5.3–40.7	7.1–14.3	
Kaempferol-3-O-glucoside	0–14.2	0.08–0.2	
Kaempferol-3-O-galactoside	-	0.05–0.2	
Isorhamnetin-3-O-rutinoside	5.3–58.0	99–191	
Isorhamnetin-3-O-glucoside	5.0–15.3	-	
Isorhamnetin-3-O-galactoside	-	3.0–7.1	
Quercetin-3-O-glucoside	0–1.7	0.5–1.6	
Quercetin-3-O-galactoside	-	3.1–12.6	
Quercetin-3-O-rutinoside	-	0.6–3.7	
**Flavanone glycosides**	**4.8–28.6**	**0.9–1.9**	
Naringenin-7-O-glucoside	2.3–25.9	0.9–1.9	
Eriodictyol-7-O-glucoside	2.5–2.7	-	
**Flavonol aglycones**	**9.6–33.0**	**1.1–4.9**	
Kaempferol	2.7–12.1	0–0.04	
Quercetin	1.0–4.9	0.2–0.3	
Isorhamnetin	5.9–16.0	0.9–4.6	
**Dihydroflavonol aglycones**	**0–10.3**	**0.4–3.0**	
Dihydroquercetin	0–10.3	-	
Dihydroxykaempferol	-	0.4–3.0	
**Flavanone aglycones**	**6.7–19.9**	**1.2–6.9**	
Naringenin	3.7–12.1	0.4–1.2	
Eriodictyol	3.0–7.8	0.8–5.7	
**Stilbenes**	**0.002**	**0.008**	**0.07–0.11**
Polydatin	1.5–2.2 ng/g	7.2–8.5 ng/g	63–84 ng/g
Piceatannol + oxyresveratrol	ND	ND	9.1–25.5 ng/g
*trans*-Resveratrol	ND	<LOD	
Pterostilbene	ND	<LOD	
**Total**	**111–418**	**132–306**	

^1^ Bold letters indicate the sum of total concentration referred to each subfamily of phenolic compounds. LOD: limit of detection; ND: not determined.

**Table 3:** Bioactivities of almond and its by-products and their mechanisms of action.

Mechanisms of Action	References
Antioxidant activity	
Free-radical scavenging activity: DPPH, ORAC, ABTS	[37,38]
Reducing power: FRAP	[37,39]
Antioxidant enzymes induction: SOD, CAT, GPx, APX	[40,41,42]
Cell antioxidant response modulation: Nrf2, ARE expression	[40]
Inhibition of lipid oxidation TBARS	[3]
Depletion of oxidative stress markers: ROS, GSH, DNA, and protein degradation	[42,43]
Anticancer activity	
Effectiveness against MCF-7, MDA-MB-468, HepG2, HCT-116, Saos-2, Colo-320, Colo-741, Caco-2, and B-16 cancer cell lines	[44,45,46,47,48]
Oxidative stress alleviation	[49]
Cell cycle arrest	[50]
Impairment of mitochondrial function and induction of caspase-mediated apoptosis	[46]
Inhibition of tumor migration, metastasis, and cell malignancy	[47]
Anti-inflammatory activity	
Inhibition of immune cell infiltration	[51]
Reduction of pro-inflammatory CKs: IL-1β, IL-6, TNF-α, CINC-1, MCP-1	[51,52]
Depletion of inflammatory mediators: PGE2, NFκB, NO, ICAM-1, selectins	[53,54,55]
Inhibition of pro-inflammatory enzyme activity: iNOS, COX-2, MPO, PARP	[51,52]
Antimicrobial activity	
Bacteriostatic effect against both pathogenic Gram-positive and Gram-negative bacteria	[39]
Antifungal activity against *C. albicans*	[39]
Antiviral activity HSV-1 and HSV-2: inhibition of viral penetration, suppression of early viral proteins and viral DNA accumulation, enhancement of antiviral immune cell response	[39]
Prebiotic activity	
Enhancement of bifidobacterial and lactobacilli populations via butyrate production	[56,57]
Promotion of β-galactosidase activity and inhibition of β-glucuronidase and azoreductase activities	[9]
Suppression of pathogenic bacteria growth	[58]

ABTS: 2,2-azino-bis(3-ethylbenzothiazoline-6-sulfonic acid); APX: ascorbate peroxidase; ARE: antioxidant response element; CAT: catalase; CINC-1: cytokine-induced neutrophil chemoattractant 1; CKs: cytokins; COX-2: cyclooxygenase 2; DPPH: 2,2-diphenyl 1-picrylhydrazyl; FRAP: ferric reducing antioxidant power; GPx: glutathione peroxidase; GSH: reduced glutathione; HSV: herpes simplex virus; ICAM-1: intercellular adhesion molecule; IL: interleukin; MCP-1: cytokine-induced neutrophil chemoattractant 1 monocyte chemoattractant protein-1; MPO: myeloperoxidase; NFκB: nuclear factor kappa B; NO: nitric oxide; Nrf2: nuclear factor-E2-related factor 2; ORAC: oxygen radical absorbance capacity; PARP: poly(ADP-ribose) polymerase; PGE2: prostaglandin E2; ROS: reactive oxygen species; SOD: superoxide dismutase; TBARS: thiobarbituric acid reactive substances; TNF-α: tumor necrosis factor alpha.

**Table 4:** Health-promoting effects of almond and its by-products on chronic diseases.

Cholesterol-Lowering and Obesity-Preventing Effects	
Reduction of TC, LDL-C, ApoB levels	[9]
Improvement of lipoprotein profile and inhibition of LDL-C oxidation	[42]
Reduction of body adiposity, body mass index, and body weight	[77]
Cardioprotective effects	
Reduction of atherogenic index	[80]
Reduction of blood pressure	[81]
Antidiabetic effects	
Reduction of blood glucose level via GLP-1 production	[82]
Reduction of carbohydrate absorption	[83]
Reduction of insulin resistance in diabetic patients	[84]
Hepatoprotective effects	
Reduction of serum ALT, AST, and GGT levels	[40,85]
Induction of liver antioxidant enzymes: SOD, GPx, CAT	[40]
Neuroprotective effects	
Alzheimer-preventing mechanisms: anxiolytic, sedative, and memory-enhancing properties	[9,86]

ALT: alanine aminotransferase; AST: aspartate aminotransferase; CAT: catalase; GGT: gamma-glutamyl transferase; GLP-1: glycoprotein 1; GPx: glutathione peroxidase; LDL-C: low-density lypoprotein cholesterol; SOD: superoxide dismutase; TC: total cholesterol.

## Data Availability

Data sharing not applicable.

## References

[1] Barreira J.C.M., Nunes M.A., da Silva B.V., Pimentel F.B., Costa A.S.G., Alvarez-Ortí M., Pardo J.E., Oliveira M.B.P.P. (2019). Almond cold-pressed oil by-product as ingredient for cookies with potential health benefits: Chemical and sensory evaluation. Food Sci. Hum. Wellness.

[2] Alasalvar C., Salvadó J.S., Ros E. (2020). Bioactives and health benefits of nuts and dried fruits. Food Chem..

[3] Esfahlan A.J., Jamei R., Esfahlan R.J. (2010). The importance of almond (*Prunus amygdalus* L.) and its by-products. Food Chem..

[4] Bartolomé B., Monagas M., Garrido I., Gómez-Cordovés C., Martín-Álvarez P.J., Lebrón-Aguilar R., Urpí-Sardà M., Llorach R., Andrés-Lacueva C. (2010). Almond (*Prunus dulcis* (Mill.) D.A. Webb) polyphenols: From chemical characterization to targeted analysis of phenolic metabolites in humans. Arch. Biochem. Biophys..

[5] Monagas M., Garrido I., Lebrón-Aguilar R., Bartolome B., Gómez-Cordovés C. (2007). Almond (*Prunus dulcis* (Mill.) D.A. Webb) skins as a potential source of bioactive polyphenols. J. Agric. Food Chem..

[6] Prgomet I., Goncalves B., Domínguez-Perles R., Pascual-Seva N., Barros A.I.R.N.A. (2017). Valorization challenges to almond residues: Phytochemical composition and functional application. Molecules.

[7] Fornés Comas J., Alonso Segura J.M., Rafel Socias i Company (2019). Shell hardness in almond: Cracking load and kernel percentage. Sci. Hortic..

[8] Lammi C., Bellumori M., Cecchi L., Bartolomei M., Bollati C., Clodoveo M.L., Corbo F., Arnoldi A., Mulinacci N. (2020). Extra virgin olive oil phenol extracts exert hypocholesterolemic effects through the modulation of the LDLR pathway: In vitro and cellular mechanism of action elucidation. Nutrients.

[9] Karimi Z., Firouzi M., Dadmehr M., Javad-Mousavi S.A., Bagheriani N., Sadeghpour O. (2021). Almond as a nutraceutical and therapeutic agent in Persian medicine and modern phytotherapy: A narrative review. Phytother. Res..

[10] Prgomet I., Gonçalves B., Domínguez-Perles R., Santos R., Saavedra M.J., Aires A., Pascual-Seva N., Barros A. (2019). Irrigation deficit turns almond by-products into a valuable source of antimicrobial (poly)phenols. Ind. Crops Prod..

[11] Moldero D., López-Bernal Á., Testi L., Lorite I.J., Fereres E., Orgaz F. (2021). Long-term almond yield response to deficit irrigation. Irrig. Sci..

[12] Aktas T., Thy P., Williams R.B., McCaffrey Z., Khatami R., Jenkins B.M. (2015). Characterization of almond processing residues from the Central Valley of California for thermal conversion. Fuel Process. Technol..

[13] Urruzola I., Robles E., Serrano L., Labidi J. (2014). Nanopaper from almond (*Prunus dulcis*) shell. Cellulose.

[14] Xie L., Bolling B.W. (2014). Characterisation of stilbenes in California almonds (*Prunus dulcis*) by UHPLC–MS. Food Chem..

[15] Summo C., Palasciano M., De Angelis D., Paradiso V.M., Caponio F., Pasqualone A. (2018). Evaluation of the chemical and nutritional characteristics of almonds (*Prunus dulcis* (Mill). D.A. Webb) as influenced by harvest time and cultivar. J. Sci. Food Agric..

[16] Bolling B.W., Dolnikowski G., Blumberg J.B., Chen C.-Y.O. (2010). Polyphenol content and antioxidant activity of California almonds depend on cultivar and harvest year. Food Chem..

[17] Barreira J.C.M., Ferreira I.C.F.R., Oliveira M.B.P.P., Pereira J.A. (2008). Antioxidant activity and bioactive compounds of ten Portuguese regional and commercial almond cultivars. Food Chem. Toxicol..

[18] Čolić S.D., Fotirić Akšić M.M., Lazarević K.B., Zec G.N., Gašić U.M., Dabić Zagorac D.Č., Natić M.M. (2017). Fatty acid and phenolic profiles of almond grown in Serbia. Food Chem..

[19] Xie L., Roto A.V., Bolling B.W. (2012). Characterization of ellagitannins, gallotannins, and bound proanthocyanidins from California almond (*Prunus dulcis*) varieties. J. Agric. Food Chem..

[20] Milbury P.E., Chen C.-Y., Dolnikowski G.G., Blumberg J.B. (2006). Determination of flavonoids and phenolics and their distribution in almonds. J. Agric. Food Chem..

[21] Beltrán Sanahuja A., Maestre Pérez S.E., Grané Teruel N., Valdés García A., Prats Moya M.S. (2021). Variability of chemical profile in almonds (*Prunus dulcis*) of different cultivars and origins. Foods.

[22] López-Ortiz C.M., Prats-Moya S., Sanahuja A.B., Maestre-Pérez S.E., Grané-Teruel N., Martín-Carratalá M.L. (2008). Comparative study of tocopherol homologue content in four almond oil cultivars during two consecutive years. J. Food Compos. Anal..

[23] Barreira J.C.M., Casal S., Ferreira I.C.F.R., Peres A.M., Pereira J.A., Oliveira M.B.P.P. (2012). Supervised chemical pattern recognition in almond (*Prunus dulcis*) Portuguese PDO cultivars: PCA- and LDA-based triennial study. J. Agric. Food Chem..

[24] EFSA Panel on Dietetic Products Nutrition and Allergies (NDA) (2011). Scientific Opinion on the Substantiation of Health Claims Related to Almonds and Maintenance of Normal Blood LDL Cholesterol Concentrations (ID 1131) and Maintenance of Normal Erectile Function (ID 2482) Pursuant to Article 13 (1) of Regulation (EC) No 19.

[25] Prats-Moya M.S., Grané-Teruel N., Berenguer-Navarro V., Martín-Carratalá M.L. (1999). A chemometric study of genotypic variation in triacylglycerol composition among selected almond cultivars. J. Am. Oil Chem. Soc..

[26] Cherif A., Sebei K., Boukhchina S., Kallel H., Belkacemi K., Arul J. (2004). Kernel fatty acid and triacylglycerol composition for three almond cultivars during maturation. J. Am. Oil Chem. Soc..

[27] Zhu Y., Wilkinson K.L., Wirthensohn M. (2017). Changes in fatty acid and tocopherol content during almond (*Prunus dulcis*, cv. Nonpareil) kernel development. Sci. Hortic..

[28] Nawade B., Yahyaa M., Reuveny H., Shaltiel-Harpaz L., Eisenbach O., Faigenboim A., Bar-Yaakov I., Holland D., Ibdah M. (2019). Profiling of volatile terpenes from almond (*Prunus dulcis*) young fruits and characterization of seven terpene synthase genes. Plant Sci..

[29] Xiao L., Lee J., Zhang G., Ebeler S.E., Wickramasinghe N., Seiber J., Mitchell A.E. (2014). HS-SPME GC/MS characterization of volatiles in raw and dry-roasted almonds (*Prunus dulcis*). Food Chem..

[30] Oliveira I., Malheiro R., Meyer A.S., Pereira J.A., Gonçalves B. (2019). Application of chemometric tools for the comparison of volatile profile from raw and roasted regional and foreign almond cultivars (Prunus dulcis). J. Food Sci. Technol..

[31] Sathe S.K., Wolf W.J., Roux K.H., Teuber S.S., Venkatachalam M., Sze-Tao K.W.C. (2002). Biochemical characterization of amandin, the major storage protein in almond (*Prunus dulcis* L.). J. Agric. Food Chem..

[32] Ahrens S., Venkatachalam M., Mistry A.M., Lapsley K., Sathe S.K. (2005). Almond (*Prunus dulcis* L.) protein quality. Plant Foods Hum. Nutr..

[33] Roncero J.M., Álvarez-Ortí M., Pardo-Giménez A., Rabadán A., Pardo J.E. (2020). Review about non-lipid components and minor fat-soluble bioactive compounds of almond kernel. Foods.

[34] De Souza T.S.P., Dias F.F.G., Oliveira J.P.S., de Moura Bell J.M.L.N., Koblitz M.G.B. (2020). Biological properties of almond proteins produced by aqueous and enzyme-assisted aqueous extraction processes from almond cake. Sci. Rep..

[35] Mandalari G., Faulks R.M., Bisignano C., Waldron K.W., Narbad A., Wickham M.S.J. (2010). In vitro evaluation of the prebiotic properties of almond skins (*Amygdalus communis* L.). FEMS Microbiol. Lett..

[36] Aguilar A.A., Smith N.E., Baldwin R.L. (1984). Nutritional value of almond hulls for dairy cows. J. Dairy Sci..

[37] Valdés A., Vidal L., Beltrán A., Canals A., Garrigós M.C. (2015). Microwave-assisted extraction of phenolic compounds from almond skin byproducts (*Prunus amygdalus*): A multivariate analysis approach. J. Agric. Food Chem..

[38] Bottone A., Masullo M., Montoro P., Pizza C., Piacente S. (2019). HR-LC-ESI-Orbitrap-MS based metabolite profiling of *Prunus dulcis* Mill. (Italian cultivars Toritto and Avola) husks and evaluation of antioxidant activity. Phytochem. Anal..

[39] Musarra-Pizzo M., Ginestra G., Smeriglio A., Pennisi R., Sciortino M.T., Mandalari G. (2019). The antimicrobial and antiviral activity of polyphenols from almond (*Prunus dulcis* L.) skin. Nutrients.

[40] Truong V.L., Bak M.J., Jun M., Kong A.N.T., Ho C.T., Jeong W.S. (2014). Antioxidant defense and hepatoprotection by procyanidins from almond (*Prunus amygdalus*) skins. J. Agric. Food Chem..

[41] Zrig A., Mohamed H.B., Tounekti T., Ahmed S.O., Khemira H. (2015). Differential responses of antioxidant enzymes in salt-stressed almond tree grown under sun and shade conditions. J. Plant Sci. Res..

[42] Chen C.Y.O., Milbury P.E., Blumberg J.B. (2019). Polyphenols in almond skins after blanching modulate plasma biomarkers of oxidative stress in healthy humans. Antioxidants.

[43] An J., Liu J., Liang Y., Ma Y., Chen C., Cheng Y., Peng P., Zhou N., Zhang R., Addy M. (2020). Characterization, bioavailability and protective effects of phenolic-rich extracts from almond hulls against pro-oxidant induced toxicity in Caco-2 cells. Food Chem..

[44] Dhingra N., Kar A., Sharma R., Bhasin S. (2017). In-vitro antioxidative potential of different fractions from *Prunus dulcis* seeds: Vis a vis antiproliferative and antibacterial activities of active compounds. S. Afr. J. Bot..

[45] Gomaa E.Z. (2013). In vitro antioxidant, antimicrobial, and antitumor activities of bitter almond and sweet apricot (*Prunus armeniaca* L.) kernels. Food Sci. Biotechnol..

[46] Khani A., Meshkini A. (2021). Anti-proliferative activity and mitochondria-dependent apoptosis induced by almond and walnut by-product in bone tumor cells. Waste Biomass Valoriz..

[47] Mericli F., Becer E., Kabadayi H., Hanoglu A., Hanoglu D.Y., Yavuz D.O., Ozek T., Vatansever S. (2017). Fatty acid composition and anticancer activity in colon carcinoma cell lines of *Prunus dulcis* seed oil. Pharm. Biol..

[48] Dammak M.I., Chakroun I., Mzoughi Z., Amamou S., Mansour H.B., Le Cerf D., Majdoub H. (2018). Characterization of polysaccharides from *Prunus amygdalus* peels: Antioxidant and antiproliferative activities. Int. J. Biol. Macromol..

[49] García-Pérez P., Barreal M.E., Rojo-De Dios L., Cameselle-Teijeiro J.F., Gallego P.P., Rahman A. (2019). Bioactive natural products from the genus Kalanchoe as cancer chemopreventive agents: A review. Studies in Natural Products Chemistry.

[50] Hikal D.M., Awad N.S., Abdein M.A. (2017). The anticancer activity of cashew (*Anacardium occidentale*) and almond (*Prunus dulcis*) kernels. Adv. Environ. Biol..

[51] Zorrilla P., Rodriguez-Nogales A., Algieri F., Garrido-Mesa N., Olivares M., Rondón D., Zarzuelo A., Utrilla M.P., Galvez J., Rodriguez-Cabezas M.E. (2014). Intestinal anti-inflammatory activity of the polyphenolic-enriched extract Amanda^®^ in the trinitrobenzenesulphonic acid model of rat colitis. J. Funct. Foods.

[52] Müller A.K., Schmölz L., Wallert M., Schubert M., Schlörmann W., Glei M., Lorkowski S. (2019). In vitro digested nut oils attenuate the lipopolysaccharide-induced inflammatory response in macrophages. Nutrients.

[53] Bisignano C., Filocamo A., La Camera E., Zummo S., Fera M.T., Mandalari G. (2013). Antibacterial activities of almond skins on cagA-positive and-negative clinical isolates of Helicobacter pylori. BMC Microbiol..

[54] Pandey P., Bhatt P.C., Rahman M., Patel D.K., Anwar F., Al-Abbasi F., Verma A., Kumar V. (2018). Preclinical renal chemo-protective potential of *Prunus amygdalus* Batsch seed coat via alteration of multiple molecular pathways. Arch. Physiol. Biochem..

[55] Mandalari G., Bisignano C., Genovese T., Mazzon E., Wickham M.S.J., Paterniti I., Cuzzocrea S. (2011). Natural almond skin reduced oxidative stress and inflammation in an experimental model of inflammatory bowel disease. Int. Immunopharmacol..

[56] Holscher H.D., Taylor A.M., Swanson K.S., Novotny J.A., Baer D.J. (2018). Almond consumption and processing affects the composition of the gastrointestinal microbiota of healthy adult men and women: A randomized controlled trial. Nutrients.

[57] Rocchetti G., Bhumireddy S.R., Giuberti G., Mandal R., Lucini L., Wishart D.S. (2019). Edible nuts deliver polyphenols and their transformation products to the large intestine: An in vitro fermentation model combining targeted/untargeted metabolomics. Food Res. Int..

[58] Liu Z., Lin X., Huang G., Zhang W., Rao P., Ni L. (2014). Prebiotic effects of almonds and almond skins on intestinal microbiota in healthy adult humans. Anaerobe.

[59] Pasqualone A., Laddomada B., Spina A., Todaro A., Guzmàn C., Summo C., Mita G., Giannone V. (2018). Almond by-products: Extraction and characterization of phenolic compounds and evaluation of their potential use in composite dough with wheat flour. LWT Food Sci. Technol..

[60] Bottone A., Montoro P., Masullo M., Pizza C., Piacente S. (2020). Metabolite profiling and antioxidant activity of the polar fraction of Italian almonds (Toritto and Avola): Analysis of seeds, skins, and blanching water. J. Pharm. Biomed. Anal..

[61] Kahlaoui M., Vecchia S.B.D., Giovine F., Kbaier H.B.H., Bouzouita N., Pereira L.B., Zeppa G. (2019). Characterization of polyphenolic compounds extracted from different varieties of almond hulls (*Prunus dulcis* L.). Antioxidants.

[62] Smeriglio A., Mandalari G., Bisignano C., Filocamo A., Barreca D., Bellocco E., Trombetta D., Karimi Z., Firouzi M., Dadmehr M. (2019). Polyphenolic content and biological properties of Avola almond (Prunus dulcis Mill. D.A. Webb) skin and its industrial byproducts. Phytother. Res..

[63] Li N., Jia X., Chen C.Y.O., Blumberg J.B., Song Y., Zhang W., Zhang X., Ma G., Chen J. (2007). Almond consumption reduces oxidative DNA damage and lipid peroxidation in male smokers. J. Nutr..

[64] Smeriglio A., Barreca D., Bellocco E., Trombetta D. (2017). Proanthocyanidins and hydrolysable tannins: Occurrence, dietary intake and pharmacological effects. Br. J. Pharmacol..

[65] Amico V., Barresi V., Condorelli D., Spatafora C., Tringali C. (2006). Antiproliferative terpenoids from almond hulls (*Prunus dulcis*): Identification and structure-activity relationships. J. Agric. Food Chem..

[66] Lim T.K. (2012). Prunus dulcis. Edible Medicinal and Non-Medicinal Plants: Volume 4, Fruits.

[67] Udenigwe C.C., Je J.Y., Cho Y.S., Yada R.Y. (2013). Almond protein hydrolysate fraction modulates the expression of proinflammatory cytokines and enzymes in activated macrophages. Food Funct..

[68] Lauro M.R., Marzocco S., Rapa S.F., Musumeci T., Giannone V., Picerno P., Aquino R.P., Puglisi G. (2020). Recycling of almond by-products for intestinal inflammation: Improvement of physical-chemical, technological and biological characteristics of a dried almond skins extract. Pharmaceutics.

[69] Barreca D., Nabavi S.M., Sureda A., Rasekhian M., Raciti R., Silva A.S., Annunziata G., Arnone A., Tenore G.C., Süntar İ. (2020). Almonds (*Prunus dulcis* Mill. D. A. webb): A source of nutrients and health-promoting compounds. Nutrients.

[70] Theophil Anand G., Renuka D., Ramesh R., Anandaraj L., John Sundaram S., Ramalingam G., Magdalane C.M., Bashir A.K.H., Maaza M., Kaviyarasu K. (2019). Green synthesis of ZnO nanoparticle using *Prunus dulcis* (Almond Gum) for antimicrobial and supercapacitor applications. Surf. Interfaces.

[71] Bouaziz F., Koubaa M., Helbert C.B., Kallel F., Driss D., Kacem I., Ghorbel R., Chaabouni S.E. (2015). Purification, structural data and biological properties of polysaccharide from *Prunus amygdalus* gum. Int. J. Food Sci. Technol..

[72] Bouaziz F., Koubaa M., Ellouz Ghorbel R., Ellouz Chaabouni S. (2017). Biological properties of water-soluble polysaccharides and hemicelluloses from almond gum. Int. J. Biol. Macromol..

[73] Bisignano C., Mandalari G., Smeriglio A., Trombetta D., Pizzo M.M., Pennisi R., Sciortino M.T. (2017). Almond skin extracts abrogate HSV-1 replication by blocking virus binding to the cell. Viruses.

[74] Arena A., Bisignano C., Stassi G., Mandalari G., Wickham M.S.J., Bisignano G. (2010). Immunomodulatory and antiviral activity of almond skins. Immunol. Lett..

[75] Mandalari G., Bisignano C., D’Arrigo M., Ginestra G., Arena A., Tomaino A., Wickham M.S.J. (2010). Antimicrobial potential of polyphenols extracted from almond skins. Lett. Appl. Microbiol..

[76] Singh R.D., Nadar C.G., Muir J., Arora A. (2019). Green and clean process to obtain low degree of polymerisation xylooligosaccharides from almond shell. J. Clean. Prod..

[77] Martins I.M., Chen Q., Oliver Chen C.Y. (2016). Emerging functional foods derived from almonds. Wild Plants, Mushrooms and Nuts: Functional Food Properties and Applications.

[78] Liu Z., Wang W., Huang G., Zhang W., Ni L. (2016). In vitro and in vivo evaluation of the prebiotic effect of raw and roasted almonds (Prunus amygdalus). J. Sci. Food Agric..

[79] Pasqualone A., Laddomada B., Boukid F., de Angelis D., Summo C. (2020). Use of almond skins to improve nutritional and functional properties of biscuits: An example of upcycling. Foods.

[80] Kalita S., Khandelwal S., Madan J., Pandya H., Sesikeran B., Krishnaswamy K. (2018). Almonds and cardiovascular health: A review. Nutrients.

[81] Eslampour E., Asbaghi O., Hadi A., Abedi S., Ghaedi E., Lazaridi A.V., Miraghajani M. (2020). The effect of almond intake on blood pressure: A systematic review and meta-analysis of randomized controlled trials. Complement. Ther. Med..

[82] Ren M., Zhang H., Qi J., Hu A., Jiang Q., Hou Y., Feng Q., Ojo O., Wang X. (2020). An almond-based low carbohydrate diet improves depression and glycometabolism in patients with type 2 diabetes through modulating gut microbiota and GLP-1: A randomized controlled trial. Nutrients.

[83] Gupta A., Sharma R., Sharma S., Nayik G.A., Gull A. (2020). Almond. Antioxidants in Vegetables and Nuts—Properties and Health Benefits.

[84] Li S.C., Liu Y.H., Liu J.F., Chang W.H., Chen C.M., Chen C.Y.O. (2011). Almond consumption improved glycemic control and lipid profiles in patients with type 2 diabetes mellitus. Metabolism.

[85] Jamshed H., Arslan J., Sultan F.T., Siddiqi H.S., Qasim M., Gilani A.-u.G. (2020). Almond protects the liver in coronary artery disease—A randomized controlled clinical trial. J. Pak. Med. Assoc..

[86] Gorji N., Moeini R., Memariani Z. (2018). Almond, hazelnut and walnut, three nuts for neuroprotection in Alzheimer’s disease: A neuropharmacological review of their bioactive constituents. Pharmacol. Res..

[87] Tungmunnithum D., Elamrani A., Abid M., Drouet S., Kiani R., Garros L., Kabra A., Addi M., Hano C. (2020). A quick, green and simple ultrasound-assisted extraction for the valorization of antioxidant phenolic acids from Moroccan almond cold-pressed oil residues. Appl. Sci..

[88] Reisman E. (2020). Superfood as spatial fix: The ascent of the almond. Agric. Hum. Values.

[89] Ryan N.T., Gradziel T.M. (2017). World almond market. Almonds: Botany, Production and Uses.

[90] Vanga S.K., Wang J., Orsat V., Raghavan V. (2020). Effect of pulsed ultrasound, a green food processing technique, on the secondary structure and in-vitro digestibility of almond milk protein. Food Res. Int..

[91] Čolić S.D., Zec G., Nati M., Fotirić Akšić M.M., Ramadan M.F. (2019). Almond (*Prunus dulcis*) oil. Fruit Oils: Chemistry and Functionality.

[92] Doulati Ardejani F., Badii K., Limaee N.Y., Shafaei S.Z., Mirhabibi A.R. (2008). Adsorption of Direct Red 80 dye from aqueous solution onto almond shells: Effect of pH, initial concentration and shell type. J. Hazard. Mater..

[93] İzgi M.S., Saka C., Baytar O., Saraçoğlu G., Şahin Ö. (2019). Preparation and characterization of activated carbon from microwave and conventional heated almond shells using phosphoric acid activation. Anal. Lett..

[94] Ait Ahsaine H., Zbair M., Anfar Z., Naciri Y., El haouti R., El Alem N., Ezahri M. (2018). Cationic dyes adsorption onto high surface area ‘almond shell’ activated carbon: Kinetics, equilibrium isotherms and surface statistical modeling. Mater. Today Chem..

[95] Pirayesh H., Khazaeian A. (2012). Using almond (Prunus amygdalus L.) shell as a bio-waste resource in wood based composite. Compos. Part B Eng..

[96] Sabbatini A., Lanari S., Santulli C., Pettinari C. (2017). Use of almond shells and rice husk as fillers of poly(methyl methacrylate) (PMMA) composites. Materials.

[97] Demirbaş A. (2002). Fuel characteristics of olive husk and walnut, hazelnut, sunflower, and almond shells. Energy Sources.

[98] Urrestarazu M., Martínez G.A., Salas M.D.C. (2005). Almond shell waste: Possible local rockwool substitute in soilless crop culture. Sci. Hortic..

[99] Krist S., Puri S. (2021). Almond oil. Vegetable Fats and Oils.

